# Routine serum biomarkers and lightgbm modeling for the diagnosis of abomasal ulcers in culled dairy cows

**DOI:** 10.1007/s11250-026-05432-9

**Published:** 2026-10-20

**Authors:** J. E. Amorim, K. G. Perestrelo, A. C. Pinheiro, J. M. N. Freitas, T. V. Q. Lancheros, C. S. Mori, F. Ferreira, M. C. A. Sucupira

**Affiliations:** 1 Department of Clinical Medicine, School of Veterinary Medicine and Animal Science, University of São Paulo, Av. Prof. Dr. Orlando Marques de Paiva 87, Cidade Universitária, São Paulo, São Paulo State 05508-270 Brazil; 2 Department of Preventive Veterinary Medicine and Animal Health, School of Veterinary Medicine and Animal Science, University of São Paulo, Av. Prof. Dr. Orlando Marques de Paiva 87, Cidade Universitária, São Paulo, São Paulo State 05508-270 Brazil

**Keywords:** Gastrin, Pepsinogen, Fecal occult blood test, LightGBM, Abomasal ulcers

## Abstract

Abomasal ulcer (AU) is a common condition in dairy cows that significantly affects productivity and longevity. This study evaluated the diagnostic value of serum biomarkers and the fecal occult blood test (FOBT), and tested a Light Gradient Boosting Machine (LightGBM) model to predict ulcer occurrence. Postmortem analyses were performed on 304 culled dairy cows, from which blood and fecal samples were collected. Animals were classified according to the presence (*n* = 249) or absence (*n* = 55) of abomasal ulcers. No individual biomarker, including gastrin or pepsinogen, demonstrated consistent diagnostic value. The FOBT showed high specificity (90.9%) but low sensitivity (27.7%), while the LightGBM model showed limited discriminatory performance (AUC ROC = 0.60). The model identified creatinine, platelet count, GGT, mean corpuscular hemoglobin, and total protein as relevant features. These findings indicate that current serum biomarkers and routine blood variables are insufficient for accurate individual diagnosis of AU, underscoring the need for more specific diagnostic tools.

## Introduction

Abomasal ulcers (AUs) are clinically relevant gastrointestinal lesions in dairy cattle, ranging from superficial mucosal ulcerations with mild or absent clinical signs to hemorrhagic or perforating ulcers associated with systemic deterioration (Braun et al. 1991b, 2020b).

AUs are particularly relevant in dairy cows and may occur more frequently during early lactation and periods of dietary change (Radostits et al. 2007). These lesions are multifactorial, with predisposing factors including high-concentrate feeding, stress, and concurrent metabolic diseases (Radostits et al. 2007).

Abomasal ulcers are commonly classified into five types (AU1–AU5), based on the system originally proposed by Whitlock (1980). Subsequent publications refined this classification by subdividing AU1 into subtypes 1A–1D and further detailing AU5 as an ulcer perforating into the omental bursa (Braun et al. 1991b, 2020c).

AU1 includes non-perforating ulcers of the abomasal mucosa and is subdivided into subtypes 1A–1D according to macroscopic appearance and lesion depth, ranging from superficial mucosal defects to deeper or more chronic ulcerative lesions, some of which may show local hemorrhage (Braun et al. 1991b, 2020a). AU2 involves erosion of a large blood vessel and is associated with massive intraluminal hemorrhage, which may lead to melena, anemia, or pale mucous membranes (Braun et al. 1991a, 2019a). AU3 and AU4 are perforating ulcers associated with localized and generalized peritonitis, respectively, whereas AU5 perforates into the omental bursa and causes omental bursitis (Braun et al. 2020b, c).

Despite these differences, ante-mortem diagnosis remains challenging because clinical manifestations may be nonspecific and single clinical variables have limited diagnostic reliability for differentiating abomasal ulcers from other gastrointestinal disorders (Braun et al. 2020b; Fecteau and Whitlock 2009).

Clinically, AUs are difficult to diagnose because many affected cattle show nonspecific manifestations such as inappetence and decreased milk yield, whereas more suggestive signs, such as melena, are mainly associated with hemorrhagic ulcers (Braun et al. 2020b). Routine hematological and biochemical analyses may reveal anemia, inflammation, or metabolic disturbances but lack diagnostic specificity (Hussain et al. 2023a). Ancillary tests, such as ultrasonography or peritoneal fluid examination, are limited to complicated cases (Gerspach et al. 2020).

Serum biomarkers, including gastrin and pepsinogen, have been proposed as potential diagnostic tools for abomasal and other gastrointestinal diseases (Hussain et al. 2024; Khalphallah et al. 2018; Mesarič et al. 2002; Ok et al. 2001). Studies have reported associations between elevated gastrin and abomasal displacement or ulceration in cattle and buffaloes (Khalphallah et al. 2018; Ok et al. 2001). Pepsinogen, a zymogen produced by abomasal chief cells, has been linked to mucosal injury and parasitic gastritis (Kataria et al. 2008; Mamak et al. 2022). However, published findings are inconsistent, possibly due to differences in animal physiology, assay techniques, and disease chronicity (Hussain et al. 2023b; Nielsen et al. 2019). The fecal occult blood test (FOBT) has also been evaluated as an indirect method to detect gastrointestinal bleeding, but its diagnostic sensitivity for abomasal ulcers remains low (Marshall 2009; Yasaswini et al. 2021).

Given these limitations, there is growing interest in integrating multiple biomarkers and computational models to improve diagnostic accuracy. Machine learning algorithms such as the Light Gradient Boosting Machine (LightGBM) offer a data-driven approach to identify complex nonlinear patterns in biochemical data (Ke et al. 2017). Therefore, the present study aimed to evaluate serum biomarkers and the FOBT for the diagnosis of AUs in culled dairy cows, and to assess the performance of a LightGBM model in predicting the presence of lesions.

## Materials and methods

### Animals and sampling

A total of 304 Holstein or Holstein-cross dairy cows were examined postmortem at a commercial abattoir in southeastern Brazil. The abattoir receives culled dairy cows from multiple farms in São Paulo State, allowing the evaluation of animals from different herd backgrounds under routine slaughter conditions. Blood samples were collected during exsanguination, and fecal samples were retrieved during evisceration. The macroscopic examination of abomasa was performed in loco at the slaughterhouse, immediately after evisceration on the processing line. The assessment was conducted in the dirty area of the facility, following standard sanitary procedures and without interfering with routine slaughter operations. Each abomasum was opened along the greater curvature and visually inspected for mucosal lesions, following the classification proposed by Braun et al. (1991b).

For anatomical localization of the lesions, the abomasum was divided into three zones according to Munch et al. (2019): Zone 1 extended from the omasoabomasal orifice to the end of the large abomasal folds; Zone 2 comprised the intermediate, relatively flat region between the large folds of Zone 1 and the smaller folds of Zone 3; and Zone 3 comprised the most aboral portion of the abomasum, including the pyloric region immediately before the duodenum.

Photographic records were taken of all observed lesions to ensure documentation and consistency in classification. Digital photographs of the lesions were analyzed using ImageJ software (Schneider et al. 2012). Each ulcer was manually outlined, and total ulcerated area was calculated for each cow as the sum of the individual ulcer areas.

Ulcer severity was assessed macroscopically based on lesion type, according to the classification system proposed by Whitlock (1980) and refined by Braun et al. (1991b, 2020b). In this classification, lesion type reflects pathological progression according to depth of tissue involvement, presence of hemorrhage, perforation, and peritoneal involvement. Ulcers were recorded as subtype 1 A (small, superficial mucosal defects), 1B (deeper, sharply demarcated defects with local hemorrhage), 1 C (crater-shaped ulcers), or 1D (ulcers with mucosal folds converging toward a central point or with holes through individual folds, without full-thickness perforation of the abomasal wall) (Braun et al. 1991b). Type 2 ulcers involve a large blood vessel and cause marked intraluminal hemorrhage. Perforated ulcers extend through the entire abomasal wall and are associated with localized peritonitis (type 3), generalized peritonitis (type 4), or omental bursitis (type 5) (Braun et al. 2020b; 2020c). Because differentiation among perforating ulcer types requires detailed assessment of peritoneal involvement during evisceration, perforating lesions were grouped as perforated ulcers under slaughterhouse workflow conditions. Ulcer type was therefore considered as a qualitative indicator of severity. In addition, ulcer burden, defined as the overall quantitative extent of ulcerative disease, was assessed using the total number of ulcers and the total ulcerated area per cow.

Because the study was conducted within the regular slaughter process, no complete necropsy examination of other organ systems was performed. Animals were categorized solely based on the presence or absence of macroscopic abomasal ulcers. Accordingly, ulcer-negative cows were defined as animals without macroscopic abomasal ulcers and were not assumed to be free of other lesions or concurrent conditions.

### Laboratory analyses

Hematological and biochemical analyses were conducted using automated equipment (model ABC Vet, ABX^®^ and Labmax 240™, Labtest^®^, respectively). Analytes included fibrinogen (Kaneko and Smith 1967), urea (BioSystems^®^, 11541), creatinine (Labtest^®^, 96–300), total protein (Labtest^®^, 99–250), albumin (Labtest^®^, 19–250), calcium (BioSystems^®^, 11570), aspartate aminotransferase (AST) (BioSystems^®^,11531), gamma glutamyl transferase (GGT) (BioSystems^®^, 11520), creatine kinase (CK) (Diasys^®^, 116019910021); and plasma beta-hydroxybutyrate (Randox^®^, RB 1007). Serum gastrin concentrations were determined using a solid-phase chemiluminescent immunometric assay (Immulite^®^ 2000xpi, Siemens^®^, 10380979) as described by Hussain et al. (2024). Serum pepsinogen concentrations were determined using the method of Paynter (1992) described by Scott et al. (1995). Fecal occult blood was detected using a benzidine-based colorimetric method, following the techniques described by Crivellenti et al. (2017) and Yasaswini et al. (2021). A parasitological analysis was performed using a modified version of the technique described by Gordon and Whitlock (1939) to rule out interference from fecal blood related to parasitic infection.

### Statistical analysis

Descriptive statistics were calculated for animal-level and lesion-level variables. Continuous variables were summarized as mean ± standard deviation or median (interquartile range), according to data distribution, whereas categorical variables were summarized as absolute and relative frequencies. Variables describing ulcer type and anatomical location were recorded at the lesion level, whereas total ulcer number and total ulcerated area were summarized per cow. Missing values resulted from unavailable or lost samples for specific laboratory analyses. Therefore, each statistical analysis was performed using all available observations for the corresponding variable. The laboratory variables evaluated included fibrinogen, gamma-glutamyl transferase (GGT), creatine kinase (CK), aspartate aminotransferase (AST), platelet count, urea, creatinine, total protein, albumin, beta-hydroxybutyrate, calcium, total leukocytes, gastrin, and pepsinogen. These variables were also considered in correlation analyses with ulcer burden quantified as total ulcer number and total ulcerated area per cow.

Continuous variables were tested for normality using the Shapiro-Wilk test. As the laboratory variables were not normally distributed, comparisons between cows with and without abomasal ulcers were performed using the Mann-Whitney test. Categorical variables were compared using the chi-square test or Fisher’s exact test when expected cell frequencies were < 5. Statistical significance was set at *P* < 0.05. No formal adjustment for multiple comparisons was applied because the laboratory variables were selected a priori based on their clinical relevance to abomasal ulceration. Accordingly, P-values were interpreted together with the magnitude and biological relevance of the observed differences. These analyses were performed in R version 4.2.2 (R Core Team 2022).

Given the non-normal distribution of the variables, associations between laboratory variables and ulcer burden (total ulcer number and total ulcerated area) were assessed using Spearman rank correlation coefficients. 95% confidence intervals for Spearman correlation coefficients were estimated using Fisher’s z transformation.

The association between fecal occult blood test (FOBT) results and the presence of abomasal ulcers was evaluated using contingency tables and the chi-square or Fisher’s exact test, as appropriate. Odds ratios were calculated, and diagnostic performance was assessed by sensitivity, specificity, positive predictive value, and negative predictive value. Diagnostic performance was also evaluated for bleeding ulcers, defined as the presence of at least one subtype 1B or type 2 ulcer. Individual ulcer subtypes were not analysed separately because biomarker measurements were obtained at the animal level, multiple ulcer types frequently coexisted in the same cow, and some ulcer categories included few affected animals, limiting the reliability of subtype-specific diagnostic estimates.

Finally, a supervised Light Gradient Boosting Machine (LightGBM) classification model was used to assess the joint predictive value of the hematological and biochemical variables for the presence of abomasal ulcers. The dataset comprised 304 cows and included 25 hematological and biochemical predictors together with a binary outcome indicating the presence or absence of abomasal ulcer. The dataset was randomly divided into training (75%) and test (25%) sets. Model training was performed using stratified five-fold cross-validation, and hyperparameters were optimized by randomized search using the area under the receiver operating characteristic curve (AUC ROC) as the optimization metric. Modeling was performed in Python 3.11 (Python Software Foundation 2022) using the pandas (McKinney 2010), scikit-learn (Pedregosa et al. 2011), and LightGBM (Ke et al. 2017) libraries.

## Results

### Descriptive findings of abomasal ulcers

A total of 304 cull dairy cows were evaluated and classified according to the presence (*n* = 249) or absence (*n* = 55) of macroscopic abomasal ulcers. Overall, 5,496 ulcers were identified in affected cows. Considering all 304 cows, including ulcer-negative animals, the mean ulcer count was 18.1 per cow, with a median of 6.5 (range, 0–349). Among cows with at least one ulcer, the mean ulcer count was 22.1 per cow, with a median of 9 (IQR, 4–23; range, 1–349).

Most ulcers occurred in Zone 1 (3,811/5,496; 69.3%), followed by Zone 3 (1,475/5,496; 26.8%) and Zone 2 (210/5,496; 3.8%). Regarding ulcer severity, subtype 1B was the most frequent lesion (3,138/5,496; 57.1%), followed by subtype 1 A (1,969/5,496; 35.8%). Subtypes 1 C and 1D accounted for 139 (2.5%) and 92 (1.7%) ulcers, respectively, whereas type 2 ulcers and perforated ulcers accounted for 73 (1.3%) and 85 (1.5%) lesions, respectively (Table 1).

**Fig. 1:**
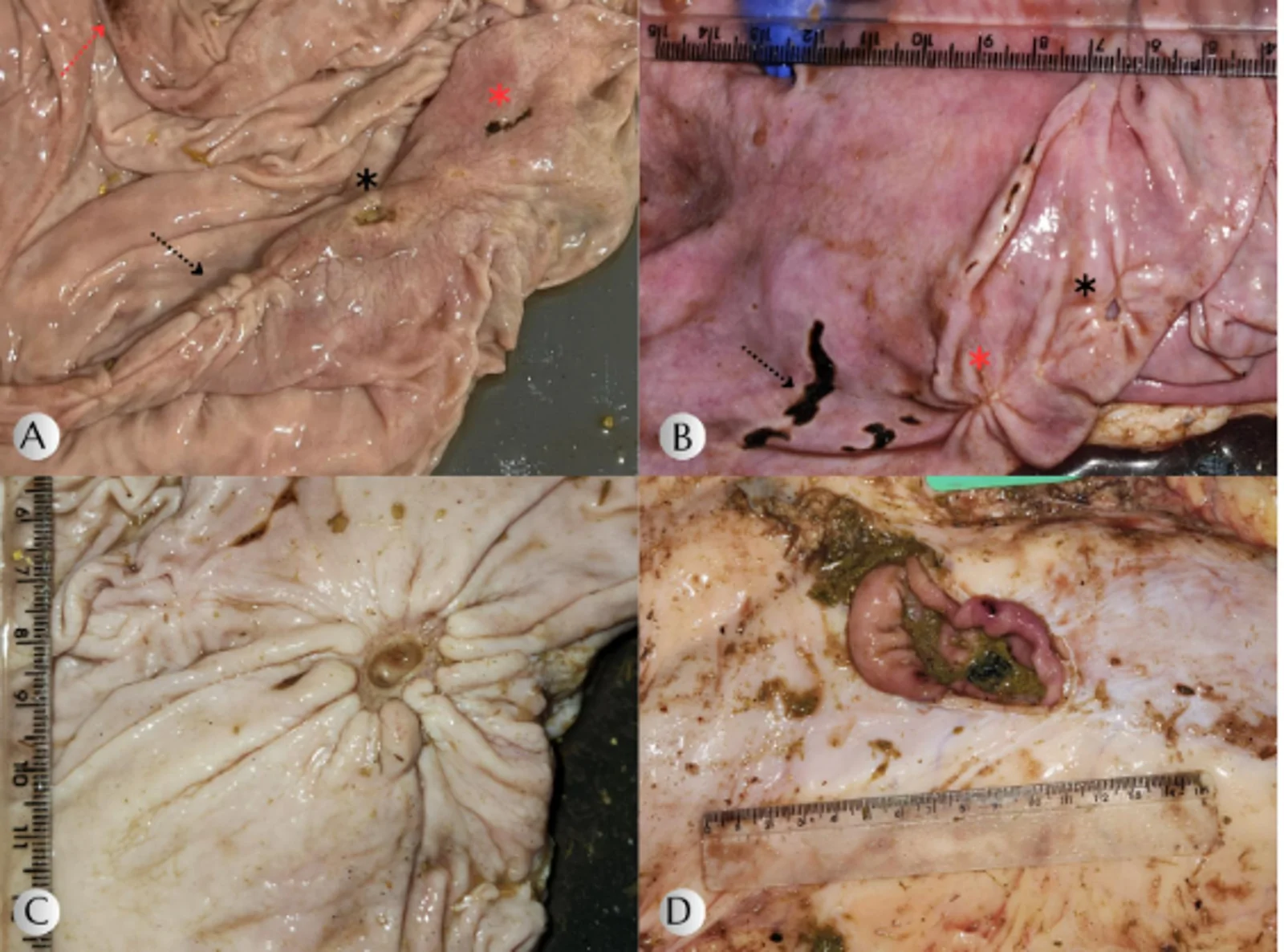
Representative macroscopic images of abomasal ulcers observed during slaughterhouse inspection. (**A**) Abomasal fold in the body/fundic region showing different ulcer types in the same area: subtype 1D ulcer on a fold (black dashed arrow), subtype 1C ulcer (black asterisk), subtype 1B ulcer (red asterisk), and subtype 1A ulcer (red dashed arrow). (**B**) Abomasal fold in the body/fundic region showing subtype 1B ulcer (black dashed arrow), perforated ulcer (black asterisk), and subtype 1D ulcer without perforation (red asterisk). (**C**) Pyloric region showing a subtype 1D ulcer, characterized by radial folds converging toward a central depressed area. (**D**) External view of the abomasum before opening, showing a perforated ulcer with abomasal content

Representative macroscopic examples of the abomasal ulcer types observed during slaughterhouse inspection are shown in Fig. 1.

**Table 1:** Distribution of abomasal ulcers according to anatomical zone and lesion type in 304 culled dairy cows

Ulcer type	Zone 1 *n*	Zone 2 *n*	Zone 3 *n*	Total ulcers *n* (%)	Cows affected *n*
1 A	1,542	23	404	1,969 (35.8)	205
1B	2,086	148	904	3,138 (57.1)	151
1 C	42	11	86	139 (2.5)	22
1D	69	16	7	92 (1.7)	38
Type 2	1	10	62	73 (1.3)	3
Perforated ulcers	71	2	12	85 (1.5)	61
Total	3,811	210	1,475	5,496 (100.0)	-

Values represent the absolute number of ulcers identified in each anatomical zone. Percentages were calculated considering the total number of ulcers recorded (*n* = 5,496). The number of affected cows indicates animals with at least one ulcer of the corresponding type; therefore, categories are not mutually exclusive because the same cow could present more than one ulcer type

### Hematological and biochemical variables

Comparisons of inflammatory, serum biochemical, and hematological variables between cows with and without abomasal ulcers are summarized in Table 2.

Body condition score did not differ between cows with and without abomasal ulcers (median 2.5, IQR 2.0–3.5 vs. median 2.5, IQR 2.0–3.0, respectively; *P* = 0.765).

Cows with abomasal ulcers had significantly higher plasma fibrinogen concentrations than ulcer-negative cows (median 400 vs. 200 mg/dL; *P* = 0.007). Gamma-glutamyl transferase (GGT) and creatine kinase (CK) activities were also significantly higher in cows with ulcers (*P* = 0.049 and *P* = 0.035, respectively).

No significant differences were observed for AST, urea, creatinine, total protein, albumin, beta-hydroxybutyrate, calcium, platelet count, total leukocyte count, gastrin, or pepsinogen (*P* > 0.05 for all variables; Table 2).

### Correlations with ulcer burden

Correlations between laboratory variables and total ulcer number are presented in Table 3.

Total ulcerated area was also evaluated as an indicator of ulcer burden; however, only plasma fibrinogen showed a weak but significant positive correlation with this variable (ρ = 0.168; *P* = 0.004), whereas no significant associations were observed for the remaining laboratory variables (*P* > 0.05).

**Table 2:** Inflammatory, serum biochemical, and hematological variables in dairy cows with and without abomasal ulcers

Variable	Unit	Ulcer group, median (min–max)*	Ulcer-negative group, median (min–max)*	*P*-value
Fibrinogen	mg/dL	400 (100–2200)	200 (200–1400)	0.007
GGT	U/L	19.7 (2.2-219.3)	16.9 (14.4-208.2)	0.049
CK	U/L	187 (10-57068)	128 (28-1890)	0.035
AST	U/L	53.3 (6.8-670.6)	45.0 (14.4-208.2)	0.051
Platelets	×10^9^/L	269 (7-992)	208 (6-616)	0.127
Urea	mmol/L	4.9 (1.6–41.8)	4.7 (1.2–27.9)	0.404
Creatinine	µmol/L	99 (57–439)	101 (66-2502)	0.825
Total protein	g/L	82.20 (31.0-106.5)	82.80 (57.1-103.1)	0.308
Albumin	g/L	27.4 (9.2–88.7)	28.3 (16.8–43.4)	0.404
Beta-hydroxybutyrate	mmol/L	0.27 (0.06–1.01)	0.25 (0.11–1.60)	0.314
Calcium	mmol/L	2.34 (1.60–3.12)	2.37 (1.80–3.96)	0.127
Total leukocytes	x10^9^/L	10.4 (4.0-60.5)	9.6 (4.5–25.2)	0.128
Gastrin	pg/mL	43.3 (10.0-923.0)	39.1 (10.0-581.0)	0.621
Pepsinogen	IU	3.4 (0.0-33.3)	2.0 (0.0–10.0)	0.168

^*^Values are expressed as median (minimum–maximum)

**Table 3:** Spearman correlations (ρ) between laboratory variables and total ulcer numbers in dairy cows with abomasal ulcers

Variable	Ulcer burden metric	*n*	Spearman ρ (95% CI)	*P*-value
Fibrinogen	Total number of ulcers	294	0.188 (0.075 to 0.296)	0.001
GGT	Total number of ulcers	290	0.117 (0.002 to 0.229)	0.047
AST	Total number of ulcers	293	0.158 (0.045 to 0.268)	0.007
Albumin	Total number of ulcers	293	-0.217 (-0.323 to -0.105)	< 0.001
Calcium	Total number of ulcers	292	-0.201 (-0.309 to -0.089)	< 0.001
Total leukocytes	Total number of ulcers	285	0.123 (0.007 to 0.236)	0.037
Gastrin	Total number of ulcers	303	-0.166 (-0.274 to -0.055)	0.003
Pepsinogen	Total number of ulcers	294	-0.063 (-0.176 to 0.052)	0.284

ρ = Spearman correlation coefficient; 95% CI = 95% confidence interval. Confidence intervals were estimated using Fisher’s z transformation

### Fecal occult blood test (FOBT)

The FOBT was performed in 297 cows because fecal samples were unavailable for seven animals.

Overall, 27.7% of cows with at least one abomasal ulcer (*n* = 67/242) tested positive, compared with 9.1% (5/55) of ulcer-negative cows (*P* = 0.0063; Fisher’s exact test), corresponding to an odds ratio of 3.83; 95% CI: 1.46–10.01. Considering the presence of any ulcer as the reference condition, the FOBT showed a sensitivity of 27.7%, specificity of 90.9%, positive predictive value of 93.1%, and negative predictive value of 22.2%.

When bleeding ulcers (at least one subtype 1B or type 2 ulcer) were considered separately, 48 of 148 cows (32.4%) tested positive, whereas 24 of 149 cows (16.1%) without these lesions were FOBT positive. This association was significant (*P* = 0.0016; odds ratio = 2.50; 95% CI: 1.43–4.36). For this outcome, the FOBT showed sensitivity of 32.4%, specificity of 83.9%, positive predictive value of 66.7%, and negative predictive value of 55.6%.

The total number of ulcers showed a weak but significant positive correlation with FOBT positivity (ρ = 0.226; *P* < 0.001).

### LightGBM model

A Light Gradient Boosting Machine (LightGBM) model was developed to evaluate the combined predictive value of hematological and biochemical variables for classifying cows according to the presence or absence of abomasal ulcers. The dataset comprised 304 cows, with missing predictor values handled natively by the LightGBM algorithm. The dataset was randomly divided into training (75%) and test (25%) sets, and model performance was evaluated on the independent test set (*n* = 76).

**Fig. 2:**
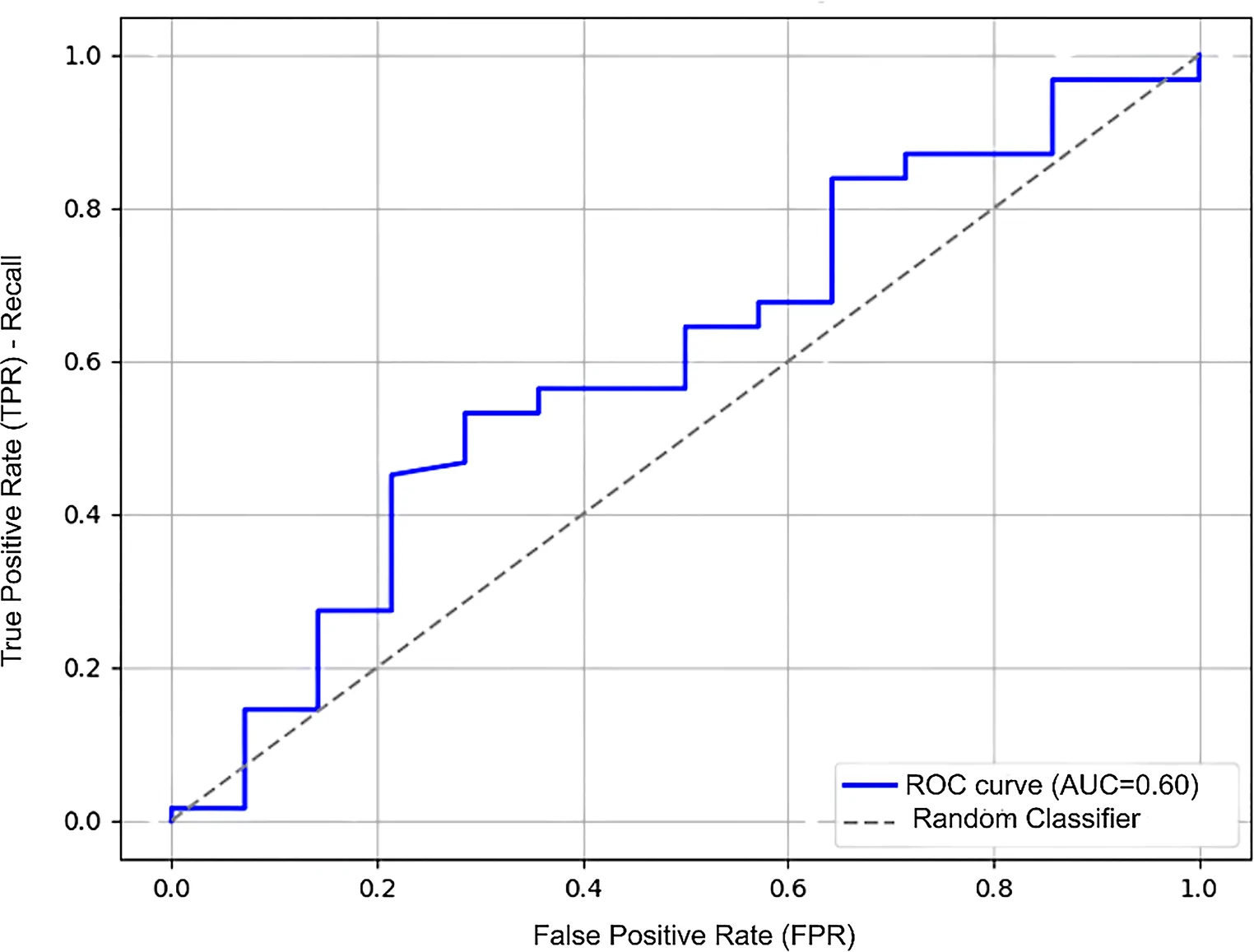
Receiver operating characteristic (ROC) curve of the Light Gradient Boosting Machine (LightGBM) model for classification of cows with and without abomasal ulcers. The dashed line represents the performance of a random classifier

The model showed limited discriminatory ability, with an area under the receiver operating characteristic curve (AUC ROC) of 0.60, which is considered poor according to published criteria (Nahm 2022). Overall classification accuracy was 61.8% (Fig. 2).

**Fig. 3:**
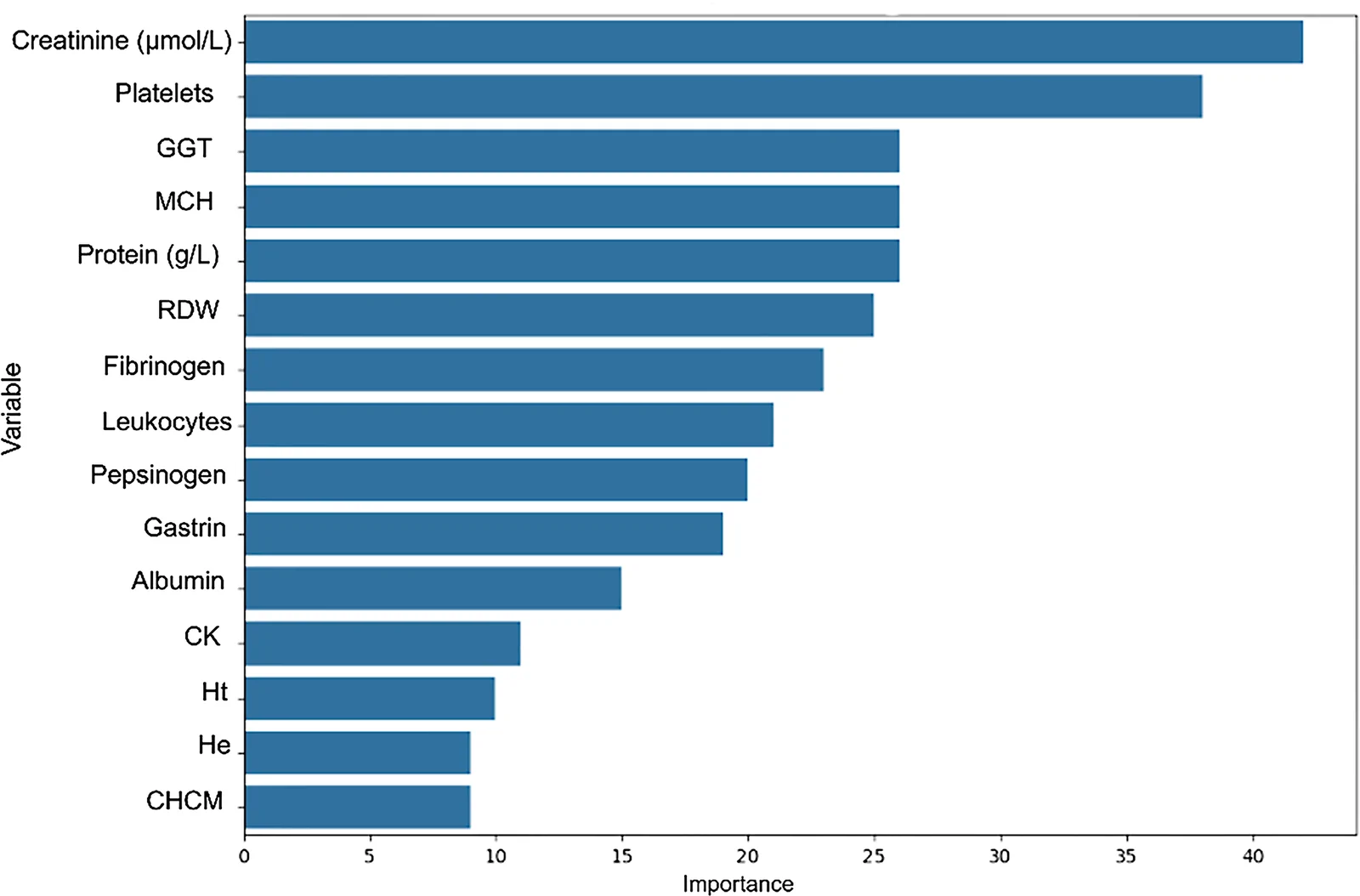
Relative feature importance (normalized gain) of the best Light Gradient Boosting Machine (LightGBM) model for predicting abomasal ulcers. Top predictors were creatinine (µmol/L), platelet count (×10⁹/L), gamma-glutamyl transferase (GGT; U/L), mean corpuscular hemoglobin (MCH; pg), and total protein (g/L)

Model performance was mainly limited by low specificity, indicating reduced ability to correctly classify cows without abomasal ulcers. Feature importance analysis based on normalized gain identified creatinine, platelet count, gamma-glutamyl transferase (GGT), mean corpuscular hemoglobin, and total protein as the variables contributing most to model predictions (Fig. 3).

## Discussion

Abomasal ulcers are widely recognized as multifactorial conditions influenced by nutritional, metabolic, infectious, and management-related factors, and are often the consequence of systemic disturbances rather than isolated gastric pathology (Radostits et al. 2007; Hussain et al. 2023a). The frequent coexistence of disorders such as ketosis, displaced abomasum, ruminal acidosis, and hepatic dysfunction compromises abomasal mucosal integrity and helps explain the limited diagnostic specificity of routine hematological and biochemical markers, which largely reflect nonspecific inflammatory or metabolic responses.

In the present study, fibrinogen showed higher concentrations in cows with ulcers and weak positive correlations with both total number of ulcers and ulcerated area, supporting its role as an acute-phase protein indicative of systemic inflammation rather than lesion-specific pathology, as previously reported by Fecteau et al. (2018). Despite this association, fibrinogen did not emerge as a relevant predictor in the multivariate LightGBM model, reinforcing its limited value as an isolated diagnostic marker, although it may still provide information regarding ulcer burden.

Hematological findings also had limited diagnostic value. Although anemia is commonly expected in animals with bleeding abomasal ulcers (Braun et al. 1991a, 2019a; Hund and Wittek 2018), packed cell volume, erythrocyte count, and hemoglobin concentrations were above reference ranges (Constable et al. 2017). This apparent relative polycythemia may reflect pre-slaughter handling, feed withdrawal, transport-related dehydration, or other non-ulcer-related factors rather than a specific marker of abomasal ulceration (Hogan et al. 2007; Gallo et al. 2003). Although total leukocyte count showed a weak positive correlation with total ulcer number, values remained mostly within normal limits, limiting its clinical usefulness as a diagnostic indicator. Higher leukocyte values in cows with perforated ulcers may reflect more severe inflammatory responses in advanced lesions, partially agreeing with reports of altered leukograms in severe cases (Tharwat and Ahmed 2012; Braun et al. 2019b; Hussain et al. 2023a).

Biochemical changes in liver and muscle enzymes were nonspecific. Although increased AST, GGT and CK activities have been reported in cattle and buffaloes with hemorrhagic abomasal ulcers (Hussain et al. 2023b), these enzymes showed limited discriminatory value in the present study. CK activity was higher in cows with ulcers, while AST showed only a weak correlation with total ulcer number and did not differ significantly between groups. GGT activity differed between groups, but showed only a weak association with ulcer burden. Together, these findings suggest that changes in these enzymes may reflect muscle damage, hepatic stress, pre-slaughter handling, or concurrent systemic conditions rather than ulcer-specific processes, especially considering the limited liver specificity of AST (Allison 2017; Constable et al. 2017; González and Silva 2006). Even considering the possible role of hepatic dysfunction and stress- related conditions in the pathogenesis of abomasal ulcers (Constable et al. 2017; Andrade Neto and Sucupira 2023; Uzal et al. 2016; Fecteau and Whitlock 2009), these enzymes did not provide consistent diagnostic discrimination in this population.

Markers related to abomasal secretory function showed limited diagnostic relevance. Gastrin, a hormone involved in gastric acid secretion and mucosal trophism (Schubert and Rehfeld 2019; Uzal et al. 2016), has been associated with abomasal displacement, ulceration, and parasitic infections in ruminants (Fox et al. 1993, 2002; Khalphallah et al. 2018). In contrast to reports of increased gastrin concentrations in cattle with hemorrhagic ulcers or in buffaloes with type 1 lesions (Ok et al. 2001; Hussain et al. 2024), gastrin concentrations in the present study did not differ between cows with and without ulcers and showed only a weak negative correlation with total number of ulcers. These findings, obtained using a chemiluminescent assay comparable to radioimmunoassay methods (Yalow and Berson 1970; Rehfeld 2021), indicate limited clinical applicability of gastrin as a diagnostic biomarker in culled dairy cows.

Similarly, pepsinogen, a precursor enzyme produced by abomasal chief cells and proposed as a marker of gastric mucosal injury (Mesarič et al. 2002; Mesarič 2005; Mamak et al. 2022), did not differ between groups and showed no association with ulcer burden. This contrasts with studies reporting increased pepsinogen concentrations in animals with hemorrhagic or perforated ulcers and in clinically affected ruminants (Hajimohammadi et al. 2017; Hussain et al. 2023c; Mamak et al. 2022; Mesarič 2005; Gouda et al. 2020). The discrepancy may be related to differences in study populations, lesion severity, clinical status, timing of sampling, and the frequent coexistence of multiple ulcer subtypes in individual cows. Because laboratory biomarkers were measured at animal level, whereas different ulcer types frequently coexisted in the same cow, a subtype-specific interpretation of serum biomarker performance would be difficult. The low number of cows with some lesion categories, particularly type 2 ulcers, further limited robust diagnostic accuracy estimates by ulcer subtype.

The fecal occult blood test was significantly associated with ulcer presence, showing high specificity but low sensitivity. Sensitivity increased modestly when only hemorrhagic ulcers were considered, supporting its use as a confirmatory tool for bleeding lesions rather than a screening method. This finding is biologically plausible because occult blood detection depends on active or recent gastrointestinal bleeding. The intermittent nature of gastrointestinal bleeding likely explains the limited sensitivity observed and suggests that repeated sampling may improve detection rates (Marshall 2009; Hajimohammadi et al. 2010). Therefore, a positive FOBT may increase suspicion of hemorrhagic abomasal ulceration, but a negative result does not exclude the presence of ulcers.

To explore whether combining multiple nonspecific variables could improve diagnostic performance, a Light Gradient Boosting Machine model was applied. The use of LightGBM was intended to evaluate whether the combined pattern of routine hematological and biochemical variables could improve prediction beyond the interpretation of individual markers. Although the model identified creatinine, platelet count, GGT, mean corpuscular hemoglobin, and total protein as the most relevant predictors, overall discriminatory performance was limited, with poor classification of cows without abomasal ulcers. This finding suggests that routine laboratory variables, even when integrated in a multivariable machine learning approach, mainly capture generalized metabolic or systemic alterations common in culled dairy cows rather than ulcer-specific processes.

This study has some limitations. Although the slaughterhouse receives culled dairy cows from various farms, individual clinical histories and detailed management information, and complete evaluations of concurrent diseases were not available. Therefore, the possible influence of unrecorded conditions on nonspecific laboratory markers cannot be excluded. In addition, the smaller number of ulcer-negative cows may have contributed to class imbalance and affected the predictive performance of the LightGBM model, particularly its specificity. Nonetheless, this slaughterhouse-based approach reflects common field conditions and allowed comprehensive post-mortem lesion assessment in a large number of culled dairy cows, together with evaluation of multiple laboratory variables.

In conclusion, routine hematological and biochemical markers showed limited diagnostic value for ante-mortem detection of abomasal ulcers in culled dairy cows. Although fibrinogen was associated with ulcer burden and fecal occult blood test showed high specificity, neither individual biomarkers nor multivariable machine learning achieved sufficient diagnostic accuracy for reliable clinical application. The FOBT may help confirm bleeding ulcers when positive but is unsuitable for broad screening because of its low sensitivity. These findings reinforce the need for more specific biomarkers and integrative diagnostic approaches beyond conventional hematological and biochemical testing.

## Data Availability

The datasets generated and analysed during the current study are stored in an institutional repository and are currently under embargo until formal publication of the manuscript. According to the institutional policy, the data may remain under restricted access for up to two years, after which they are expected to become publicly available. In the meantime, the datasets are available from the corresponding author on reasonable request.
